# Semen quality and frozen semen production in Pasundan bulls: A molecular weight perspective on seminal plasma and spermatozoa protein

**DOI:** 10.5455/javar.2023.j728

**Published:** 2023-12-31

**Authors:** Abdullah Baharun, Annisa Rahmi, Ristika Handarini, Tulus Maulana, Syahruddin Said, Hikmayani Iskandar, Imam Darussalam, Wilmientje Marlene Mesang Nalley, Raden Iis Arifiantini

**Affiliations:** 1 Department of Animals Science, Faculty of Agriculture, Djuanda University, Bogor, Indonesia; 2 Research Center for Applied Zoology, National Research and Innovation Agency, Bogor, Indonesia; 3 Technical Unit of Beef Cattle Breeding and Artificial Insemination Development Ciamis, West Java Province Departement of Food Security and Livestock, Ciamis, Indonesia; 4 Faculty of Animal Husbandry, Marine and Fisheries, Universitas Nusa Cendana, Kupang, Indonesia; 5 Division of Veterinary Reproduction and Obstetrics, School of Veterinary Medicine and Biomedical Sciences, IPB University, Bogor, Indonesia

**Keywords:** Fresh semen, local bulls, Pasundan bull, sperm quality, West Java bulls

## Abstract

**Objective:**

To determine the correlation between the molecular weight (MW) of proteins in seminal plasma and spermatozoa and the quality of fresh and frozen semen production in Pasundan bulls.

**Materials and methods:**

Nine selected Pasundan bulls, aged 5–10 years, from the Regional Artificial Insemination Center at Ciamis, West Java, Indonesia, were used in the study, with fresh semen sperm motility ≥70% and <70%. We analyzed the motility, viability, integrity of the intact plasma membrane (IPM), and the morphological characteristics of spermatozoa. 1D-SDS-PAGE analysis was performed to determine the protein profile by assessing MW, depicted as bands on the gel.

**Results:**

The motility, viability, and IPM of spermatozoa had lower values (*p* < 0.05) in Pasundan bulls named Bagaskara and Kertarajasa compared to the other bulls. Proteins with MW 35–50 kDa were not detected in the seminal plasma of Pasundan bulls, exhibiting low quality in fresh semen. The correlation analysis showed that the non-detected proteins with MW 35–50 kDa in seminal plasma correlated with spermatozoa motility (*r =* 0.421), viability (*r =* 0.424), and IPM (*r =* 0.428) so that fresh semen quality was low in both Pasundan bulls. Analysis of semen volume, spermatozoa concentration, and spermatozoa motility showed that the average frozen semen production of Pasundan bulls per ejaculate was 128.73 ± 15.35 straws.

**Conclusion:**

Protein analysis based on MW is a predictive indicator for the quality of fresh semen and the production of frozen semen in Pasundan bulls. Evaluation parameters of fresh semen quality by MW analysis can be used to select Pasundan bulls in Indonesia.

## Introduction

The uniqueness of Indonesia’s local livestock genetic resources (LLGR) must be preserved to meet national meat demand and accelerate recovery from the foot and mouth disease outbreak. One of the potential LLGR resources is Pasundan bull, which is the local bull germplasm of West Java, according to Minister of Agriculture Decree No. 1051/kpts/SR.120/10/2014 [1]. The provincial government of West Java has initiated the improvement of genetic quality through the SEPATU village innovation program (Pasundan cattle) using selected Pasundan bull breeds. Pasundan bulls were selected based on a breeding soundness examination (BSE), which, among other criteria, evaluates the quality of fresh semen as an essential parameter for the production of frozen semen in artificial insemination (AI) programs.

The quality of fresh semen from Pasundan bulls was conventionally evaluated, resulting in semen from selected males showing a relatively low fertility efficiency of less than 60%. Semen quality analysis in the BSE program includes parameters such as mass movement, spermatozoa motility, spermatozoa concentration, and spermatozoa morphology [2]. However, conventional semen analysis parameters still allow selected Pasundan bull studs to show the quality of fresh and frozen semen that does not meet the Indonesian National Standard (SNI number: 4869.1: 2021 for frozen bovine semen) [3], which may affect fertility. Male fertility is determined by intrinsic and extrinsic factors in both seminal plasma and spermatozoa. Male fertility can be affected by molecular factors, specifically the intricate interaction between the composition of seminal plasma and spermatozoa. These elements play a complex role in modulating the fertilization process, including their potential to determine vital functions in the reproductive process [4]. Several parameters that support the reproductive process, including cell protection, capacitation, acrosome response, oocyte activation, sperm motility, fertilization, and embryonic development, can be determined by functional proteins [5].

Proteins within seminal plasma play a vital role in controlling the protection of sperm [6], and this regulation may be associated with high or low male fertility. Druart and de Graaf [7] reported that fertility-related proteins can be expressed in both seminal plasma and spermatozoa. Protein expression in seminal plasma and spermatozoa can be assessed by their molecular weight (MW), which is associated with sperm function such as motility, abnormalities, viability, and intact plasma membrane (IPM) in Bali bulls and with frozen semen production [8]. However, an evaluation of fresh semen quality based on MW in Pasundan bull has never been reported. This study aims to evaluate the MW of seminal plasma protein and spermatozoa concerning fresh semen quality and the potential to produce frozen semen, serving as an additional indicator for the selection process of Pasundan bulls in Indonesia.

## Materials and Methods

### Ethical approval and experimental animals

The materials used in this study were fresh semen from Pasundan bulls obtained from the Regional Artificial Insemination Center (RAIC) in Ciamis, West Java. The study’s animal models and experimental designs were approved by the Animal Ethics Commission at the School of Veterinary Medicine and Biomedical Science, IPB University, under certificate number 067/KEH/SKE/VII/2023. The bulls at RAIC were managed following standard operational procedures supervised by a veterinarian, fulfilling every animal welfare principle.

Nine selected Pasundan bulls, aged between 5 and 10 years, from the West Java RAIC at Ciamis, West Java, Indonesia, were used in the study. Pasundan bulls were fed with a daily ration comprising 10% of their body weight (BW) in fresh forage and 1% of their BW in concentrate, both provided in the morning and evening. Water was available to them at all times. The study utilized Pasundan bulls, categorizing them based on sperm motility as either equal to or greater than 70% or less than 70%. Semen collection occurred throughout the period from January to December 2021.

### Semen collection and evaluation

The semen of Pasundan bulls was regularly collected twice a week in the morning using an artificial vagina. The collected semen was transported to the laboratory for both macroscopic and microscopic analyses. Macroscopic analysis encompassed the measurement of semen volume, color, consistency, and acidity (pH). Microscopic analysis included evaluating sperm motility through computer-assisted spermatozoa analysis, utilizing equipment maintained at a constant temperature of 37°C, and employing the Andro Vision Program (Minitub^®^, Tiefenbach, Germany). Motility movements were categorized as total motility, while the viability and morphology of spermatozoa were determined using eosin-nigrosin staining. Mixing 20 µl of semen with 80 µl of eosin-nigrosin dyes on a slide glass, and subsequently examine the mixture using a microscope (Olympus^®^ CX43) at a magnification of 400×. The concentration of spermatozoa was determined with the Photometer SDM 6 (Minitub^®^, Tiefenbach, Germany). The integrity of the plasma membrane (IPM) was evaluated through the hypo-osmotic swelling test, via the combination of 20 µl of semen with 1 ml of hypo-osmotic medium (0.7 gm sodium citrate and 0.3 gm fructose in 100 ml distilled water), followed by a 30-min incubation in a water bath at 37°C.

### Determination of fresh semen protein concentration and SDS-PAGE analysis

The fresh semen was washed three times using phosphate buffered saline (PBS) and centrifuged at 1,800 rpm. Following the manual’s instructions, the spermatozoa pellet was extracted using PRO-PREP^TM^ protein extraction solution (iNtRON Biotechnologi, Korea). The total soluble protein concentration of the sample was measured before analysis by SDS-PAGE. The colorimetric detection and quantification of total protein were conducted using the bicinchoninic acid (BCA) method, employing the Pierce™ BCA Protein Assay Kit 23225 from Thermo Scientific™, USA. SDS-PAGE analysis was performed to determine the protein profile based on MW, which is represented as bands on the gels. Protein separation was carried out using SDS-PAGE using SurePAGE™, Bis-Tris, 10 × 8 cm, 12 wells, 4%–20% gradient gel (M00656; GenScript) (SurePAGE, Genscript Biotech Corp. Hongkong) with Broad Multi Color Pre-Stained Protein Standard (M00624; GenScript) with a MW range of ~5–270 kDa and Tris-MOPS-SDS Running Buffer (M00138; GenScript) with a voltage of 140 V and a current of 75 mA for 55 min. The gel was then stained using InstantBlue^®^ Coomassie Protein Stain (ab119211; abcam). The differential intensity of individual protein bands was assessed by conducting ratio analysis with the aid of ImageJ software [9].

### Statistical analysis

The data on fresh semen quality were analyzed using a descriptive method. The obtained results presented the mean ± standard error for all parameters. The correlation between semen characteristics, seminal plasma, and sperm protein was analyzed with Pearson’s correlation (Sigma Plot software version 14.0). A one-way ANOVA (SPSS software 26 version, IBM^®^ Corp., Armonk, NY) was applied to the data.

## Results and Discussion

### Pasundan bulls: fresh semen quality and frozen semen production

The fresh semen quality of nine Pasundan bulls showed an average semen volume of 6.2 ml, a pH of 6.4, a milky-white color, and medium consistency. The analysis results demonstrated a notable difference in the quality of fresh semen among Pasundan bulls, particularly those named Bagaskara and Kertarajasa. The parameters of motility (36.97% ± 3.53% and 52.75% ± 2.14%), viability (51.62% ± 4.03% and 63.08% ± 2.01%), and IPM of spermatozoa (64.71% ± 3.41% and 72.43% ± 1.34%) indicated significantly lower values (*p <* 0.05) for these bulls compared to their counterparts (Table 1). The low quality of fresh semen in both bulls ensured that the fresh semen was not suitable for processing into frozen semen according to Regulation No. 10/Permentan/PK.210/3/2016 [10] by the Indonesian Minister of Agriculture and Indonesian National Standard (SNI) 4868.1:2021 regarding frozen semen production, where the minimum motility of fresh semen is ≥70%. Gallo et al. [11] reported that low spermatozoa motility in Pasundan bulls (Bagaskara and Kartarajasa) was associated with the production of adenosine triphosphate (ATP) for sperm flagellum movement. Sperm motility relies on the availability of energy generated through the ATP hydrolysis process, glycolysis, and oxidative phosphorylation (OXPHOS) for ATP production in the microtubules of the axoneme [12]. The average results of sperm motility, viability, and MPU parameter analysis in this study were lower than those described by Santoso et al. [13] in Pasundan bulls, which were at 79.72%, 82.38%, and 88.32%, respectively. A decrease in mitochondrial activity may lead to a reduction in the functionality of OXPHOS metabolism, resulting in low spermatozoa motility [14]. Mitochondria are vital for a range of cellular functions, including generating energy, regulating apoptosis, and maintaining calcium balance. In the male reproductive system, these organelles are pivotal in the formation and maintenance of germ cells, significantly impacting the production of vigorous and healthy sperm [14]. Reactive oxidative stress (ROS) can become unbalanced as a result of mitochondrial physiological dysfunction, which can have a negative effect on spermatozoa motility.

Arif et al. [15] reported that the percentage of viable sperm that can be processed into frozen sperm is 64–80%. The findings of this study indicate that the sperm viability in the semen of Pasundan bulls with Bagaskara and Kartarajasa ID (Table 1) does not meet the standards for processing into frozen semen or used in AI. The results of this study are lower than the viability of fresh semen from Bali bulls (81.30%) [16]. The low viability of spermatozoa from Pasundan bulls (Bagaskara and Kartarajasa) can be attributed to variations in the structure and ultrastructure of sperm components, which influence the strength of the plasma fluid of the spermatozoa membrane, which differs between individual bulls [16].

**Table 1.** The quality of the fresh semen from Pasundan bull.

Bulls ID	Sperm motility (%)	Spermatozoa viability (%)	Spermatozoa abnormality (%)	Spermatozoa IPM (%)
Bagaskara	36.97 ± 3.53^b^	51.62 ± 4.03^b^	6.69 ± 0.45^a^	64.71 ± 3.41^b^
Kertarajasa	52.75 ± 2.14^c^	63.08 ± 2.01^c^	7.73 ± 0.45^a^	72.43 ± 1.34^c^
Cakrabuana	71.70 ± 1.29^a^	82.18 ± 1.27^a^	5.83 ± 0.27^a^	84.60 ± 1.13^a^
Ranggasakti	77.92 ± 0.94^a^	87.63 ± 0.93^a^	6.25 ± 0.36^a^	88.44 ± 1.12^a^
Bratasena	73.83 ± 0.98^a^	83.79 ± 1.19^a^	4.89 ± 0.24^a^	84.03 ± 1.10^a^
Sanjaya	72.55 ± 1.63^a^	83.77 ± 2.12^a^	5.50 ± 0.40^a^	83.30 ± 2.34^a^
Purbasora	70.78 ± 1.67^a^	78.03 ± 1.86^a^	7.59 ± 0.49^a^	83.76 ± 2.36^a^
Sastrawinata	76.04 ± 1.91^a^	84.96 ± 1.92^a^	4.86 ± 0.37^a^	86.75 ± 1.74^a^
Anggapraja	70.38 ± 1.61^a^	77.70 ± 2.02^a^	4.94 ± 0.48^a^	78.24 ± 2.61^a^
Means ± SE	66.99 ± 1.74	76.97 ± 1.92	6.03 ± 0.39	80.69 ± 1.90

Means in a column with different superscripts a, b, and c differ significantly at *p <* 0.05.

The abnormalities of spermatozoa from all Pasundan bulls in this study did not show different results (*p* > 0.05) (Table 1). The morphological evaluation of spermatozoa abnormalities is crucial for determining fertilization success and spermatozoa-ovum capacitation; this pertains to the capacity of spermatozoa to traverse the zona pellucida [17]. The results of whole plasma membrane analysis showed that Pasundan bulls named Bagaskara and Kartarajasa had lower values (*p* < 0.05) compared to other males (Table 1). Baharun et al. [18] reported that an IPM plays an essential role in metabolic processes associated with spermatozoa motility and viability, and it is essential for fertilization, fertility, and spermatozoa function for successful AI. Impairment to the plasma membrane can diminish sperm motility, alter spermatozoa morphology and metabolism, and cause an imbalance of intracellular components such that spermatozoa fertility may be compromised.

**Figure 1.**
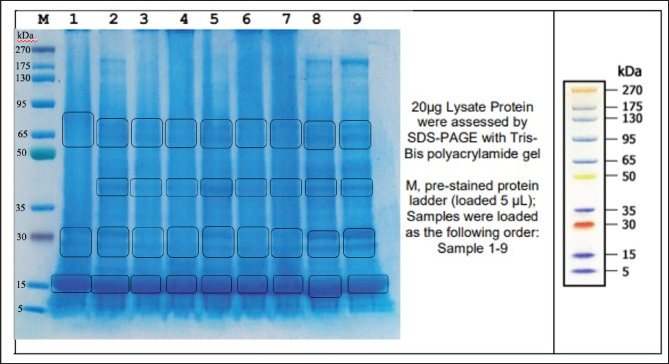
The protein profile of the seminal plasma of Pasundan Bulls. kDa: kilodaltons; M: marker; 1: Bagaskara; 2: Kertarajasa; 3: Cakrabuana; 4: Ranggasakti; 5: Bratasena; 6: Sanjaya; 7: Purbasora; 8: Sastrawinata; 9: Anggapraja.

In addition to conducting an analysis of the quality of fresh semen, the assessment of frozen semen production holds significance as it directly correlates with the quantification of frozen semen straws derived from each Pasundan bull. The semen production potential of Pasundan bull is determined by the number of ejaculations per year, corresponding to a production period of 40 weeks or 80 collections (assuming two collections per week). An analysis of semen volume, spermatozoa concentration, and spermatozoa motility showed that the average frozen semen production of Pasundan bulls per ejaculate was 128.73 ± 15.35 straws (Table 2). Frozen semen production per ejaculate was less than what was reported by Santoso et al. [13] in Pasundan bulls (167.73 straws) and in Bali cattle (225.52 straws) [8]. The reduced production of frozen semen (straws) in this study may be related to the low quality of fresh semen (motility and concentration of sperm) in Pasundan bulls with ID Bagaskara and Kartarajasa (Table 2), which is associated with the non-expression of proteins with MW 35–50 kDa in seminal plasma (Fig. 1). Seminal plasma comprises protein components and other elements like lipids, organic acids, enzymes, and minerals. These constituents play a vital role in spermatozoa metabolism and could influence the fertility of spermatozoa [19]. The expression of proteins in seminal plasma with MWs of 35–50 kDa may negatively affect spermatozoa fertility. Baharun et al. [19] reported that protein expression in seminal plasma may either negatively or positively correlate with spermatozoa fertility or spermatozoa quality.

### The MW of proteins in seminal plasma and spermatozoa of Pasundan bulls

**Figure 2.**
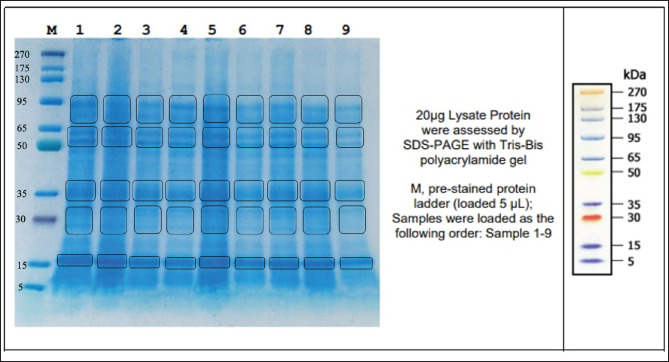
The protein profile of the spermatozoa of Pasundan Bulls. kDa: kilodaltons; M: marker; 1: Bagaskara; 2: Kertarajasa; 3: Cakrabuana; 4: Ranggasakti; 5: Bratasena; 6: Sanjaya; 7: Purbasora; 8: Sastrawinata; 9: Anggapraja.

**Figure 3.**
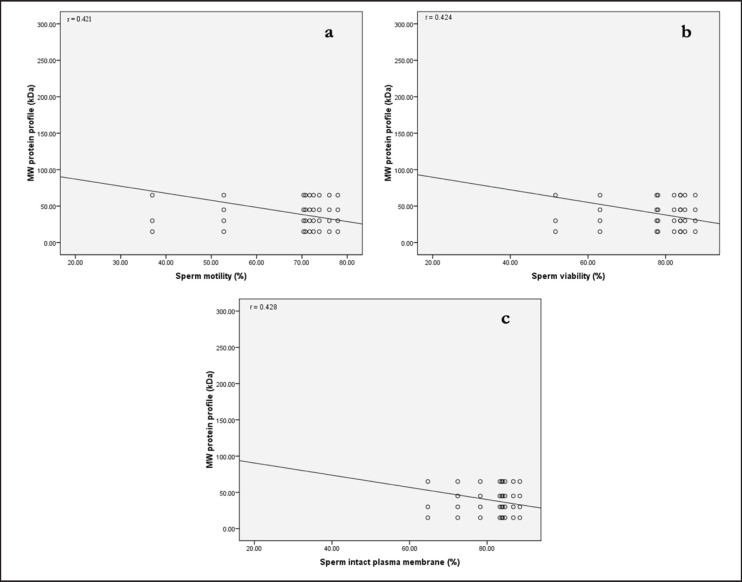
Correlation MW between fresh semen quality: correlation between spermatozoa motility (a), viability (b), and spermatozoa IPM (c) with the protein profile (kDa) of the seminal plasma of Pasundan bulls.

The results showed that a total of three plasma protein bands with MWs of 15 kDa, 30 kDa, and 65 kDa, respectively, were expressed in all bulls (Fig. 1). The expression of protein bands in fresh semen samples had MW ranging of 15 kDa, 30 kDa, 35 kDa, 50-65 kDa, and 95 kDa in all males (Fig. 2). Proteins with an MW of 15–30 kDa are referred to as binder sperm proteins (BSPs), which are expressed in seminal plasma and interact with Pasundan bovine sperm. BSPs can be divided into BSP A1/A2, BSP-A3, and BSP-30 (BSP1, BSP3, and BSP5). Rego et al. [20] reported that 60% of GNPs can be found in *Bos taurus* and *Bos indicus* seminal plasma. GNP proteins (GNP1, GNP3, and GNP5) can interact with sperm-adhesin-1 protein (SPADH1) and affect sperm motility by activating the cGMP-PKC pathway via PKC binding with CNP ligand (2’,3’-cyclic nucleotide 3’-phosphodiesterase) with NPR-B receptor (guanylate cyclase). This activation process leads to the conversion of ATP into cyclic adenosine monophosphate, which is important for sperm movement. It was found that proteins with MW 35–50 kDa were not expressed in the seminal plasma of Pasundan cattle with the name Bagaskara (Fig. 1) but were expressed in the protein bands of sperm samples (Fig. 2). The analysis showed a negative correlation between the quality of fresh semen from Pasundan bulls and the identified semen protein bands exhibiting various MWs, motility (*r =* 0.421), viability (*r =* 0.424), and IPM (*r =* 0.428). The *r*-values indicate a moderate correlation, as shown in Figure 3.

**Table 2.** The productivity of the frozen semen of Pasundan bull.

Bull ID	Semen volume (ml)	Fresh semen motility (%)	Spermatozoa concentration (×10^6^/ml^−1^)	Total motile sperm/ejaculate	Total straw/ejaculate
Bagaskara	5.93 ± 0.13	36.97 ± 3.53	720.17 ± 20.05	1,578.84	63.15
Kertarajasa	4.02 ± 0.16	52.75 ± 2.14	785.25 ± 83.16	1,665.16	66.61
Cakrabuana	5.28 ± 0.25	71.70 ± 1.29	901.00 ± 76.93	3,410.97	136.44
Ranggasakti	5.97 ± 0.46	77.92 ± 0.94	877.46 ± 32.86	4,081.79	163.27
Bratasena	6.39 ± 0.22	73.83 ± 0.98	1,102.15 ± 57.76	5,199.65	207.99
Sanjaya	5.73 ± 0.22	72.55 ± 1.63	806.83 ± 56.64	3,354.09	134.16
Purbasora	6.12 ± 0.64	70.78 ± 1.67	719.89 ± 70.10	3,118.37	124.73
Sastrawinata	6.30 ± 0.54	76.04 ± 1.91	813.67 ± 31.18	3,897.90	155.92
Anggapraja	5.92 ± 0.32	70.38 ± 1.61	637.60 ± 61.13	2,656.56	106.26
Means ± SE	5.74 ± 0.37	66.99 ± 1.74	818.22 ± 54.42	3,218.15 ± 383.95	128.73 ± 15.35

The low motility and IPM of spermatozoa in the fresh semen of Pasundan Bagaskara bulls could be attributed to the absence of proteins with MWs of 35–50 kDa in the seminal plasma. This result agrees with the finding reported by Azizah et al. [21] that the region of proteins with a MW of 45 kDa is positively correlated with motility and IPM of spermatozoa in Madura bull. Moreover, the non-expression of proteins with a MW of 35–50 kD may be associated with glutathione peroxidase 3 (GPX3), which could be associated with low spermatozoa viability. The interaction between GPX3 and sulfhydryl oxidase 1 (QSOX1) plays a vital role in how sperm responds to oxidative stress by involving cyclic GMP (cGMP). Cyclic GMP serves as an intracellular component that can modulate nitric oxide and natriuretic peptides, regulating various biological processes [21] and contributing to the maintenance of intact spermatozoa plasma membranes. The low viability and IPM (Bagaskara and Kartarajasa males) are associated with sperm plasma membrane damage due to the generation of oxidative stress through the cGMP mechanism. Increased intracellular cGMP signals physiology responses through two cyclic pathways: the cGMP-protein kinase G (PKG), cGMP-regulated phosphodiesterases (PDE2, PDE3), and cGMP-gated cation channels mediated by PKG-substrate-specific activation of PKG1 result in decreased cytosolic calcium concentration and decreased sensitivity of myofilaments to Ca^2+^ desensitization. Most biological processes regulated by intracellular Ca receptors, including calmodulin [22], can activate various types of enzymes, such as protein kinases, phosphatases, and phosphodiesterase [30]. Na/K-ATPase and inositol-1,4,5-triphosphate receptors influence the increased intracellular Ca concentration in the prostate [22]. Ca concentrations in the prostate, seminal vesicles, and epididymis may be associated with sperm infertility [22]. Na/K-ATPase plays an essential role in maintaining membrane potential (Em) and electrochemical gradients (Na^+^ and K^+^) across the sperm membrane, which is related to the structure and ultrastructure of the spermatozoa membrane [23].

The expression of proteins with a MW of 50–65 kDa in seminal plasma and spermatozoa can indicate proteins that play a role in the function of catalytic activity, such as regulating energy metabolism in mitochondria [24]. Proteins like mitochondrial sirtuin 5 (SIRT5) may influence male fertility by regulating mitochondrial function and overseeing oxidative stress. This involves the removal of ROS produced during mitochondrial metabolism [25]. SIRT5 affects spermatozoa motility, acrosome integrity, and mitochondrial Em, essential parameters for sperm fertility [26]. Furthermore, proteins with an MW of 50–65 kDa in spermatozoa could include the IZUMO1 protein located in the acrosomal membrane. IZUMO1 can interact with JUNO proteins found in oocytes, and this interaction is essential for the fertilization process [27].

The analysis showed that the sperm protein expressed in all Pasundan bulls with an MW of 95 kDa (Fig. 2) could be identified as arylsulfatase-A, which was found in the head of spermatozoa. Arysulfatase-A may be associated with the low abnormal spermatozoa morphology in all Pasundan cattle in this study (Table 1). Spermatozoa morphology is a crucial aspect for evaluating the fertility of Pasundan bulls. Protein expression plays a role in protecting spermatozoa structure and function during spermatogenesis, reducing abnormal morphology. Abnormalities in spermatozoa may arise from disruptions occurring in either the process of spermiogenesis or during maturation in the epididymis [28]. Abnormal structures in spermatozoa may be related to motility mediated by the expression and interaction of proteins from both seminal plasma and spermatozoa [29]. The MW-based protein profiles in seminal plasma and spermatozoa require further confirmation to ensure that specific proteins in fresh semen can be employed as fertility markers so that they become the basis for selecting excellent Pasundan breeding animals. Specific protein confirmation methods can use liquid chromatography-mass spectrometry (LC-MS/MS).

## Conclusion

Protein analysis based on MW can be utilized to predict the quality of fresh semen and frozen semen production in Pasundan bulls. Evaluation parameters of fresh semen quality by MW analysis (SDS-PAGE) can be employed for the selection of Pasundan bulls in Indonesia. Further analysis is required to confirm specific protein MW more accurately in fresh semen quality determination by the LC-MS/MS method (proteomics analysis).

## List of abbreviations

ATP, adenosine triphosphate; BSE, breeding soundness examination; BSPs, binder sperm protein; BW, body weight; Em, membrane potential; GPX3, glutathione peroxidase 3; LC-MS/MS, liquid chromatography-mass spectrometry; MW, molecular weight; OXPHOS, oxidative phosphorylation; QSOX1, sulfhydryl oxidase 1; RAIC, Regional Artificial Insemination Center; ROS, reactive oxidative stress; SDS-PAGE, sodium dodecyl-sulfate polyacrylamide gel electrophoresis; SIRT5, mitochondrial sirtuin5 SPADH1, spermadhesin 1.
